# Effects of simulation-based education for neonatal resuscitation on medical students’ technical and non-technical skills

**DOI:** 10.1371/journal.pone.0278575

**Published:** 2022-12-01

**Authors:** Jiwon Lee, Jang Hoon Lee

**Affiliations:** 1 Office of Medical Education, Ajou University School of Medicine, Suwon, Gyeonggi-do, Republic of Korea; 2 Department of Pediatrics, Ajou University School of Medicine, Suwon, Gyeonggi-do, Republic of Korea; King Saud bin Abdulaziz University for Health Sciences, SAUDI ARABIA

## Abstract

Simulation is a learner-centered practice that helps develop and maintain knowledge, skills, and competencies. This study evaluated the effect of neonatal resuscitation simulation-based education for medical students in the fifth year (part of the regular clinical clerkship program) on the perceived performance of their technical and non-technical skills. In addition, we analyzed the difference between instructor’s and learners’ evaluations of technical skills after the simulation. A one-group pretest-posttest design was adopted. The simulation-based education of the neonatal resuscitation program (NRP) was conducted for 40 medical students from July to November 2020 at a medical school in South Korea. The simulation-based education comprised 5 minutes of pre-briefing, 10 minutes of running the simulation, and 30 minutes of debriefing (using a recorded video). The perceived performance of students’ technical and non-technical skills before and after the simulation was compared by collecting and analyzing the pre- and post-questionnaires. The perceived performance of technical (*p* = .001) and non-technical skills (*p* < .001) was found to have significantly increased after the simulation. Particularly, the performance of technical skills, such as diagnostic (*p* = .007) and therapeutic actions (*p* < .001) and non-technical skills, such as leadership (*p* < .001), teamwork (*p* = .001), and task management (*p* = .020) improved significantly. There was no significant difference in the evaluations of the technical performance of the instructor and learners after the simulation (*p* = .953). Simulation-based education can improve technical skills, such as diagnostic and therapeutic actions for neonatal resuscitation. It is also effective in enhancing non-technical skills, such as leadership, teamwork, and task management. Further, after the simulation-based education, students can fully self-evaluate through objective reflection and improve their clinical competency.

## Introduction

Simulation-based medical education is learner-centered and experiential, being useful for achieving educational objectives and improving students’ knowledge, skills, and abilities. It also facilitates the acquisition of clinical skills through repeated practice, thereby improving patient safety and reducing medical costs [1, 2].

Then major clinical competencies of medical students include history-taking, professional attitude, physical examination, diagnostic skills, therapeutic skills, resuscitation skills, critical thinking, problem-solving skills, teamwork, and communication skills [3]. Simulation-based education (SBE) is particularly effective in crisis resource management and helps students acquire clinical skills effectively in a safe environment [4]. It thus contributes to the improvement of technical skills (TS) and non-technical skills (NTS), such as interprofessional communication, teamwork, situation awareness, task management, leadership, and decision-making [5, 6]. The improvement of NTS can further enhance medical students’ clinical competencies [7]. Previous studies have found that simulation enabled students to make clinical decisions rapidly based on priorities in emergencies, which improves their performance [8]. Therefore, the importance of SBE for medical students has increased.

Trained personnel are required to proficiently perform neonatal resuscitation, which is an essential emergency medical technique in high-risk situations. This has been made possible through simulation-based repetitive training [9, 10]. The main technical aspects of a neonatal resuscitation program (NRP) include intermittent positive pressure ventilation (IPPV), intubation, chest compression, and medication administration [11]. Simulation is an extremely effective educational method that integrates theoretical and practical knowledge in a risk-free environment [10]. Learning TS and NTS such as teamwork and leadership through simulation-based team training enables students to perform professional tasks through information-sharing and appropriate role assignment, ultimately contributing to reducing the resuscitation time of newborns [10, 12].

By debriefing after the simulation, learners can increase their knowledge and performance through self-reflection [13, 14]. Although studies on effective debriefing techniques are ongoing and various methods have been hitherto suggested, further research on the effectiveness of learner-led debriefing is needed [14–16]. To determine the effectiveness of learner-led debriefing, it is necessary to first determine whether students can objectively evaluate themselves.

Therefore, this study evaluated the effects of SBE for neonatal resuscitation on medical students’ TS and NTS perceived performance. Additionally, we analyzed the difference between the instructor’s and learners’ evaluations of TS after the simulation to confirm whether students could objectively self-evaluate their skills.

## Materials and methods

### Study design

A one-group pretest-posttest design was used to investigate the effects of NRP simulation-based education.

### Participants

We evaluated the effects of SBE as part of a clinical practice curriculum conducted for all fifth-year medical students at Ajou University in Gyeonggi-do, South Korea. The curriculum for medical school is six years long. Fifth-year medical students experience first-time clinical practicums. They first practice in internal medicine, followed by surgery, obstetrics and gynecology, pediatrics, and psychiatry. A total of 40 students responded to the questionnaire (29 males and 11 females). The mean age of the participants was 24.55 years.

### Ethical considerations

This study was conducted with the approval of the Institutional Review Board (IRB) of Ajou University Medical Center in Korea on February 03, 2021 (IRB Protocol No. AJIRB-SBR-SUR-20-589). At the beginning of the clerkship, comprehensive consent was obtained from students regarding the study of the curriculum. This study was conducted to confirm the educational effects of a SBE implemented in a regular clinical clerkship program. Along with the SBE, the students participated in pre/post surveys. The study was performed using retrospective research based on the survey data obtained from the participating students; therefore, written consent was exempted and the anonymized data were analyzed.

### Procedure

NRP simulation-based education was conducted as part of a pediatric clinical practice course for eight students (one cycle: four teams, two students per team). From July to November 2020, all 40 students (20 teams) completed the simulation through five cycles. One week before performing the SBE, eight students were provided with pre-training on the NRP for two hours by a pediatrician. Before the pre-training, medical students performed self-directed learning by watching instructional videos on neonatal resuscitation overview, techniques, and teamwork. During the pre-training, they practiced skills using the task trainer and a simulator that was powered off, received feedback from a pediatrician, and learned the application of the algorithm while watching neonatal resuscitation videos by the American Heart Association (AHA) guidelines (NRP 7^th^ edition). The pediatrician then explained to the students the difference between adult and neonatal resuscitation. The SBE was conducted the following week.

A hybrid simulation was performed by integrating a preterm simulator (Laerdal Premature Anne), in which vital signs were changed by a computer program that caused physiological reactions, such as cyanosis, for an infant tracheal intubation model (a task trainer). Hybrid simulation refers to a curriculum that combines two or more simulation modalities [17].

The SBE on the NRP comprised 5 minutes of pre-briefing (medical equipment confirmation and simulator and scenario introduction: a premature infant born by cesarean section weighing 1,500 g at 32 weeks and 2 days of gestation), 10 minutes of running the simulation, and 30 minutes of debriefing (using a recorded video). During the pre-briefing, the students were provided with learning objectives and a brief description of neonatal resuscitation by the professor. The learning objectives of this simulation education were to have students learn the NRP algorithm, perform assessments and treatments of newborns immediately after delivery, and perform major techniques such as IPPV, intubation, and chest compression based on clinical judgment. It was also to learn NTS such as communication and collaboration among medical staff in emergencies. The information about the mother and fetus in the scenario was as follows. The mother had had a cesarean section because her blood pressure was not controlled, owing to preeclampsia. The newborn was a high-risk premature infant and had just been delivered to the operating room. The newborn was a boy, showed gasping breathing with weak crying, and had poor muscle tone. A professor of pediatrics performed a simulation evaluation using the tool for evaluating TS and an operator with experience in simulation education performed a high-fidelity simulator adjustment. The newborn’s vital signs and status changes were controlled using Simpad (a portable computer linked to the simulator), and the changed vital signs were transmitted to the patient monitor for the students to check. Heart rate and percutaneous oxygen saturation were automatically displayed on the patient monitor according to the instructor’s judgment, and students were provided information on respiration and cyanosis as answers to their questions. In addition, the students participated in the simulation practice in pairs (one leader and one member) and the advanced practice nurse (APN) working at the newborn intensive care unit (NICU) participated as an embedded participant. The students performed history taking using pre-birth questions and a primary assessment (gestation, tone, crying) of the newborn. After performing the initial steps (warm and maintain temperature, position airway, clear secretions, stimulate), they selected and performed appropriate resuscitation techniques such as IPPV, intubation, chest compression, and medication administration based on the continuous assessment of the heart rate and respiration. All procedures were recorded on video. The team leader communicated with the team members regarding the role assignment and procedures to be performed. The embedded participant provided the necessary items for IPPV, preparations for intubation according to the needs of the students, and information about the simulation running time. She also prepared the medication as requested by the students, which the students administered to the newborn through the endotracheal tube.

For each SBE cycle, four teams (eight students) participated in the simulation on the same day. After the simulation running, debriefing was conducted twice sequentially on the same day. Each debriefing session was conducted for two teams (four students) over 30 minutes. A total of four students conducted debriefing while watching two simulation videos recorded with a professor in a separate debriefing room. These were self-simulation performance videos for self-monitoring. As a facilitator, the professor encouraged the students to reflect on their actions and talk about what they felt, what they did well, and what difficulties they encountered during the simulation. In addition, the facilitator provided constructive feedback and guided them to discuss future applications in similar clinical situations. Simultaneously, the four students not involved in debriefing discussed freely among themselves the clinical situation and their performance in another classroom. After 30 minutes, the four students in the debriefing room and the four students in the classroom switched places and performed the same processes as above.

### Data collection

From July 24 to November 20, 2020, the professor conducted a total of 20 simulations of neonatal resuscitation for 40 medical students (20 teams, two students per team), once per team. Prior to the simulation practice, a pretest for TS and NTS was conducted. During the simulation, the instructor evaluated TS. Afterward, each student conducted a posttest on TS and NTS to evaluate their performance and the instructor performed a simulation debriefing.

### Data analysis

Data were analyzed via nonparametric statistical analysis using IBM SPSS (version 25.0) and Jamovi (version 1.6.23). Certain variables did not satisfy the normality assumption (Shapiro–Wilk test, p < .05). The effectiveness before and after the SBE for students’ TS and NTS was compared using the Wilcoxon signed-rank test. The Mann–Whitney U test and Bland–Altman plot were used to test the difference between the instructor’s evaluation and the learners’ self-evaluation of their TS.

### Measures

#### 1) Technical skills

To evaluate the TS through simulation training, Queen’s simulation assessment tool (QSAT) [18] was used. Based on the neonatal resuscitation algorithm [19], the tool was modified and used according to the advice of a professor of pediatrics at Ajou University Medical Center. It comprised three subdomains: (1) primary assessment, (2) diagnostic actions, and (3) therapeutic actions. The TS was evaluated on a five-point Likert scale: 1 = *Inferior*, 2 = *Novice*, 3 = *Competent*, 4 = *Advanced*, and 5 = *Superior*. The total score range was 3–15, with higher scores indicating higher technical performance.

For the pretest, TS were evaluated as an individual’s perceived performance, with one item per subdomain. For the posttest, TS were evaluated based on the perceived performance regarding the items in each subdomain as follows: (1) primary assessment (three items): history taking of the newborn and mother, equipment preparation, assessment of the newborn (term gestation, good tone, breathing, or crying); (2) diagnostic actions (three items): cardiopulmonary status (apnea/gasping, chest movement, heart rate, and saturation/electrocardiogram (ECG) monitoring); and (3) therapeutic actions (seven items): airway opening, temperature maintenance, clear airway, IPPV, chest compression, intubation, and medication administration (inject the correct dose of epinephrine through the correct route; endotracheal tube). To objectively evaluate the TS of the learners, each checklist item was evaluated as 0 = *not performing*, 1 = *inaccurate performance*, or 2 = *accurate performance*. In comparison with the TS for the pretest, we converted the TS scores using a five-point Likert scale according to the sum of the scores for each subdomain, and analyzed the effectiveness of the pretest and posttest. Cronbach’s α value for the scale was 0.81.

#### 2) Non-technical skills

To evaluate NTS through simulation training, they were assessed using the TEAM instrument developed by Cooper et al. [20] and translated into Korean [21]. It comprised three subdomains with 11 items in total, including two on leadership, seven on teamwork, and two on task management. NTS were evaluated on a five-point Likert scale ranging from 0 to 4. The total score ranged from 0 to 44, with higher scores indicating high non-technical performance. For the pretest, NTS were evaluated from 0 = *cannot do it* to 4 = *can do it very well*, while for the posttest, scores ranged from 0 = *never performed* to 4 = *always performed*.

Cronbach’s α values for the scale in the original and present study were 0.92 and 0.95, respectively.

## Results

### Effects of the NRP simulation on TS

The perceived performance of the TS of the students increased significantly from a mean of 3.26 (**±**0.56) to 3.84 (**±**0.79) after the simulation (*p* < .001). In particular, among the subdomains, the TS performance of diagnostic (*p* = .007) and therapeutic actions (*p* < .001) increased significantly. Diagnostic actions increased from a mean of 3.28 (**±**0.60) to 3.88 (**±**1.02), and therapeutic actions from a mean of 3.28 (**±**0.72) to 4.38 (**±**0.87) (Table 1).

**Table 1:** Effects of neonatal resuscitation program simulation on technical skills. (n = 40).

Area	Mean ± SD		*t*	*p*
	Pretest	Posttest		
**Technical skills**	3.26 ± 0.56	3.84 ± 0.79	−3.37	.001
Primary assessment	3.23 ± 0.62	3.28 ± 1.15	−0.46	.644
Diagnostic actions	3.28 ± 0.60	3.88 ± 1.02	−2.71	.007
Therapeutic actions	3.28 ± 0.72	4.38 ± 0.87	−4.34	< .001

### Effects of NRP simulation on NTS

The perceived performance of the NTS of the students increased significantly from a mean of 2.52 (**±**0.54) to 2.91 (**±**0.66) after the simulation (*p* < .001). Among the subdomains of NTS performance, leadership (*p* < .001), teamwork (*p* = .001), and task management (*p* = .020) all increased significantly. Leadership increased from a mean of 2.43 (**±**0.65) to 2.95 (**±**0.68), teamwork from a mean of 2.52 (**±**0.53) to 2.89 (**±**0.69), and task management from a mean of 2.61 (**±**0.65) to 2.93 (**±**0.78) (Table 2).

**Table 2:** Effects of neonatal resuscitation program simulation on non-technical skills. (n = 40).

Area	Mean ± SD		*t*	*p*
	Pretest	Posttest		
**Non-technical skills**	2.52 ± 0.54	2.91 ± 0.66	−3.65	< .001
Leadership	2.43 ± 0.65	2.95 ± 0.68	−4.07	< .001
Teamwork	2.52 ± 0.53	2.89 ± 0.69	−3.45	.001
Task management	2.61 ± 0.65	2.93 ± 0.78	−2.32	.020

### Differences between the TS evaluations of the instructor and learners

There was no significant difference in the evaluations of the perceived performance of TS between the learners and the instructor. However, for the TS performance in the subdomain of diagnostic actions, learners’ self-assessment mean score of 3.88 (**±**1.02) was significantly higher than the instructor’s mean score of 3.35 (**±**1.21) (*p* = .048) (Table 3).

**Table 3:** Differences between the evaluations of technical skills of the instructor and learners.

Area	Mean ± SD		*t*	*p*
	Instructor (n = 40)	Learner (n = 40)		
**Technical skills**	3.73 ± 0.92	3.84 ± 0.79	−0.06	.953
Primary assessment	3.70 ± 1.24	3.27 ± 1.15	−1.82	.069
Diagnostic actions	3.35 ± 1.21	3.88 ± 1.02	−1.98	.048
Therapeutic actions	4.15 ± 1.12	4.38 ± 0.87	−0.71	.478

**Fig 1:**
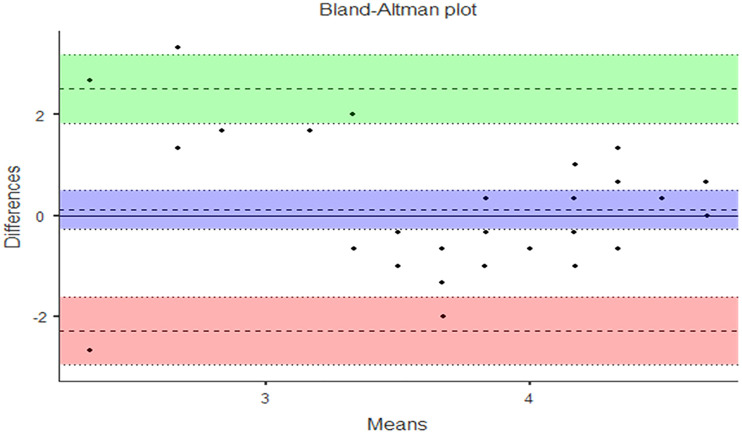
Differences in the evaluation of technical skills between the instructor and learners.

To confirm that learners’ self-evaluation was a measurement of objective performance, we analyzed the Bland–Altman plot of the differences between the learners’ and instructor’s evaluations after the simulation. The TS scores of the raters were concentrated within the 95% confidence interval, except for one data point, indicating that the agreement between the learners and the instructor regarding the TS performance evaluation was high. Moreover, the higher was the performance evaluation score, the smaller was the difference between raters (Fig 1).

## Discussion

Through the NRP simulation-based education, medical students’ perceived performance of TS improved. In particular, diagnostic actions (respiration, chest movement check, and ECG/saturation monitoring) for the newborn and accurate therapeutic actions such as IPPV/positive end-expiratory pressure ventilation, chest compression, and medication administration were improved. In a previous study, SBE increased the resuscitation score, allowed critical actions, and achieved fast resuscitation steps [22]. However, the primary assessment displayed the lowest score for TS and did not significantly improve after the simulation. Therefore, it was considered it would be difficult for students to prepare and check the equipment for neonatal resuscitation in an actual emergency or to understand the initial state of the child, such as term/muscle tone/breathing or crying; it is thus necessary to strengthen prior education on the primary assessment of whether routine care or ongoing evaluation is needed according to pre-birth questions.

Through the NRP simulation-based education, medical students’ perceived performance of NTS also improved. An integral part of the NRP requires teamwork and psychomotor skills [23]. SBE was shown to improve TS performance and affects NTS performance, such as situation awareness, decision-making, communication, teamwork, leadership, managing stress, and coping with fatigue [6, 24]. Similarly, the perceived performance improved, particularly in terms of leadership and teamwork. This indicated that, in an emergency, medical students improved their ability to monitor the situation and properly allocate the necessary interventions to cope with the situation among team members through effective communication. Even in previous studies, a high-fidelity resuscitation simulation training program improved learners’ knowledge, self-confidence, teamwork, leadership, situation monitoring, mutual support, communication skills, and critical behaviors for neonatal resuscitation [9, 25, 26].

In this study, medical students’ perceived performance of TS and NTS was improved by applying timely interventions in collaboration with the team members. SBE, which includes repetitive learning, individual feedback, and various difficulty adjustments, helps students make decisions based on priorities during emergencies [27]. The experience of simulation during clerkship can satisfy students’ learning needs, relieve their anxiety, and enhance their interest and confidence in their performance skills [28, 29]. Learners who experienced SBE responded to emergencies, such as cardiac arrest effectively, with better protocol compliance [30]. In particular, medical students who experienced simulation using a high-fidelity simulator performed resuscitation that more closely followed the AHA guidelines. Similarly, simulation practice helps students learn the basic principles, techniques, and communication between medical staff and flexibly respond to high-pressure and high-risk emergencies with professional knowledge [31]. SBE was effective in teaching the management of neonatal resuscitation; learners’ performance and confidence increased after training and were maintained even after six weeks in a short-term follow-up [32]. However, in the case of long-term follow-up (more than 6 months) after the SBE, there may be a deterioration in learners’ performance. Therefore, to develop actual clinical competence, it is important to continuously consolidate the acquired knowledge and clinical skills into practice and strengthen confidence [33].

Finally, to verify learners’ objective self-evaluation on TS, a posttest was conducted after the simulation and before debriefing to exclude the influence of the professor’s comments on students’ self-evaluation during debriefing. When comparing the evaluation of the TS performance between learners and instructors, there was no significant difference. It appears that students could objectively reflect on their actions. It can be considered that self- or peer-led debriefing instead of instructor-led debriefing may be useful during the SBE. In previous studies related to crisis SBE, peer-led debriefing improved clinical performance as much as instructor-led debriefing did [15]. In addition, it has been demonstrated that self-debriefing is cost-effective and can improve teamwork by enabling SBE while reducing the number of instructors [34]. Therefore, peer-led debriefing may achieve an effect similar to instructor-led debriefing. For effective peer-led debriefing, discussions and the self-reflection of learners by watching simulation recordings during debriefing are very important. Moreover, the self-evaluation and peer evaluation through a structured checklist can help objective self-understanding. However, in this study, the students overestimated their diagnostic actions. This could be because the APN participated as an embedded participant of the resuscitation team during the simulation and assisted in diagnostic actions such as ECG monitoring, pulse oximetry, and auscultation of the heart rate. The importance of ECG monitoring is particularly emphasized in the NRP 8^th^ edition when alternative airways are needed [35]. In a previous study targeting residents, SBE improved their accuracy in auscultating heart rates [36]. Therefore, it is necessary to enable students to improve their accuracy by performing diagnostic actions themselves during simulation practice.

Deliberate simulation practice and video-based debriefings can improve students’ neonatal resuscitation skills and shorten the time for effective spontaneous breathing from real neonatal resuscitations [37]. It was confirmed that SBE for NRP reduced the gap in the medical students’ perceived performance of TS and NTS. Furthermore, it can be considered that students’ clinical competence will ultimately improve if they experience a real emergency through clinical practice and apply the empirical knowledge and skills gained from SBE.

### Limitations

A limitation of this study is that it is difficult to generalize the effects and understand the long-term effects of SBE because the NRP module was conducted with only a few students in a single medical school and a one-group pre-posttest design was adopted. In addition, the QSAT tool may have limitations in measuring TS because it has been modified and used by a pediatric professor according to the NRP algorithm.

This study is meaningful, in that SBE reduced the gap in medical students’ perceived TS and NTS performance. However, it was not possible to exactly verify whether their TS and NTS clinical performance improved after the simulation because the perceived performance competence in the pretest and the students’ actual performance in the posttest were compared. To improve students’ problem-solving ability in emergencies, we could not inform them regarding the clinical situation in the simulation in advance, so the measurement of their actual performance in the pretest was replaced with that of their perceived performance. Therefore, to allow the actual TS and NTS performance to be measured in the future, the study design needs further consideration. In addition, during the simulation running, vital sign monitoring was possible by adjusting the physiological state with a tablet PC; however, it was difficult to perfectly reproduce reality because muscle tone, skin color, and cyanosis could not be directly observed in the simulator. In addition, although the NRP recommends intravenous or intraosseous routes for drug administration, the umbilical venous catheter could not be reproduced in the simulator. Therefore, the students administered the drugs through an endotracheal tube.

As this study was conducted in 2020, it is based on the NRP 7^th^ edition. However, since January 2022, the NRP 8^th^ edition by AHA has been implemented, in which the pre-birth questions (add umbilical cord management plan), realignment of initial steps, emphasis on cardiac monitoring, changes in epinephrine dose, and expanded timeframe for cessation of resuscitative efforts were changed. In the future, education and evaluation reflecting these aspects should be conducted.

In the current clinical curriculum of the medical school in this study, in addition to pediatric simulations, simulation training using high-fidelity simulators (respiratory system disorders, digestive system disorders, and gynecological diseases) has been repeatedly implemented to strengthen student competence in emergencies. We thus propose a time-series study to track the long-term effects of SBM on coping with emergencies for medical students and pediatric residents at various institutions.

## Conclusions

Simulation-based medical education that can be repeatedly practiced in a safe environment and controlling its various levels of difficulty contributes to enhancing medical students’ perceived performance of TS related to diagnosis and treatment and NTS such as leadership, teamwork, and task management.

During debriefing, students are fully capable of self-evaluation through objective reflection. As such, the application of learner-centered debriefing methods such as self-debriefing and peer-led debriefing can improve medical students’ clinical performance, communication skills, and confidence in a safe environment.

## Supporting information

S1 Appendix Evaluation form for technical skills (before and after simulation). (PDF)

S2 Appendix Comparison of changes in non-technical skills after simulation practice (Items). (PDF)
