# Supplementary material for: Effects of simulation-based education for neonatal resuscitation on medical students’ technical and non-technical skills
Source: PLoS One. 2022 Dec 1;17(12):e0278575. doi: 10.1371/journal.pone.0278575 (PMC9714940; doi:10.1371/journal.pone.0278575)
Supplement: S1 Appendix — (PDF) [file pone.0278575.s001.pdf]

Evaluation form for technical skills before simulation practice

1) Technical Skills						
**Category**	**Competence**	**Inferior**	**Novice**	**Competent**	**Advanced**	**Superior**
**Primary Assessment**	After delivery of the newborn, can you find out the history taking and the current condition?	①	②	③	④	⑤
**Diagnostic Actions**	Can you accurately diagnose the newborn's condition?	①	②	③	④	⑤
**Therapeutic Actions**	Can you establish appropriate care plans according to the change of the newborn's condition, and implement them?	①	②	③	④	⑤

Evaluation form for technical skills after simulation practice*

1) Technical Skills

Category	Competence	not performing	performance	
			inaccurate	accurate
Primary Assessment	history taking of the newborn and mother	⓪	①	②
	equipment preparation and provision of a safe environment for neonatal resuscitation	⓪	①	②
	assessment for the newborn (gestation, muscle tone, breathing, crying)	⓪	①	②
Diagnostic Actions	check apnea/gasping, chest movement	⓪	①	②
	heart rate, & saturation monitoring	⓪	①	②
	ECG monitoring	⓪	①	②
Therapeutic Actions	position airway, warm and temperature maintenance	⓪	①	②
	suction the nose and mouth (clear secretions)	⓪	①	②
	providing positive-pressure ventilation (PPV) (HR below 100/min: mask fit, 21% oxygen, PIP 20-25cmH_2O, PEEP 5cmH_2O 40-60/min)	⓪	①	②
	Heart rate monitoring within 15 seconds after providing PPV (continuous PPV, HR below 100/min)	⓪	①	②
	Intubation, perform chest compression at the correct location and the appropriate depth	⓪	①	②
	3 times chest compression and 1 times ventilation at an appropriate rate	⓪	①	②
	medication (right drug, dose, route) (epinephrine[1:10000], Intravenous 0.01~0.03mg/kg or endotracheal 0.05~0.1mg/kg every 3-5min)	⓪	①	②

※ Sum up the scores for each area and mark the appropriate criteria below.

1) Primary Assessment

Inferior(≤2)	Novice(3)	Competent(4)	Advanced(5)	Superior(6)
①	②	③	④	⑤

2) Diagnostic Actions

Inferior(≤2)	Novice(3)	Competent(4)	Advanced(5)	Superior(6)
①	②	③	④	⑤

3) Therapeutic Actions

Inferior(≤3)	Novice(4-6)	Competent(7-8)	Advanced(9-11)	Superior(≥12)
①	②	③	④	⑤

* This form was used for instructor's evaluation and students' self-evaluation for their technical skills
