# Supplementary material for: Effects of simulation-based education for neonatal resuscitation on medical students’ technical and non-technical skills
Source: PLoS One. 2022 Dec 1;17(12):e0278575. doi: 10.1371/journal.pone.0278575 (PMC9714940; doi:10.1371/journal.pone.0278575)
Supplement: S2 Appendix — (PDF) [file pone.0278575.s002.pdf]

S2 appendix. Comparison of changes in nontechnical skills after simulation practice (Items)

Area	Item	Mean ± SD		*t* (pre-post)
		Pretest	Posttest	
Leadership	The team leader let the team know what was expected of them through direction and command	2.45±0.71	2.95±0.64	-4.42***
	The team leader maintained a global perspective (monitoring clinical procedures and the environment, task delegation)	2.40±0.71	2.95±0.85	-4.11***
	**Sub total**	2.43 ± 0.65	2.95 ± 0.68	−4.07***
Teamwork	The team communicated effectively (verbal, non-verbal forms of communication)	2.68±0.73	2.85±0.83	-1.55
	The team worked together to complete tasks in a timely manner	2.45±0.68	2.95±0.88	-3.49***
	The team acted with composure and control (applicable emotions, conflict management)	2.40±0.78	2.88±0.76	-3.32**
	The team morale was positive (appropriate support, confidence, spirit, optimism, determination)	2.58±0.64	3.03±0.8	-3.64***
	The team adapted to changing situations (adaptation within the roles of profession, situation change)	2.50±0.68	2.88±0.79	-3.06**
	The team monitored and reassessed the situation	2.50±0.64	2.85±0.8	-2.21*
	The team anticipated potential actions (preparation of drugs, airway equipment)	2.53±0.78	2.78±0.89	-1.61
	**Sub total**	2.52 ± 0.53	2.89 ± 0.69	−3.45**
Task management	The team prioritized tasks	2.53±0.72	2.93±0.83	-2.81**
	The team followed approved standards/guidelines (Perform according to neonatal resuscitation guideline)	2.70±0.65	2.93±0.83	-1.55
	**Sub total**	2.61 ± 0.65	2.93 ± 0.78	−2.32*
**Total**		2.52 ± 0.54	2.91 ± 0.66	−3.65***

$^{*}p < .05$, $^{**}p < .01$, $^{***}p < .001$

[For each item, we evaluated pretest ranged from 0 (“can’t do it”) to 4 (“can do very well”), and posttest from 0 (“never performed”) to 4 (“always performed”)]
